# Classification of African Native Plant Foods Based on Their Processing Levels

**DOI:** 10.3389/fnut.2022.825690

**Published:** 2022-04-29

**Authors:** Afam I. O. Jideani, Oluwatoyin O. Onipe, Shonisani E. Ramashia

**Affiliations:** ^1^Vicfame Pty Ltd., Cape Town, South Africa; ^2^Special Interest Group, Postharvest Handling Group, ISEKI-Food Association, Vienna, Austria; ^3^Department of Food Science and Technology, Faculty of Science Engineering and Agriculture, University of Venda, Thohoyandou, South Africa

**Keywords:** exotic fruits, ultra-processing, cereals, tuber, minimal processing

## Abstract

With increasing advocacy for plant food consumption, the sub-Saharan Africa landscape is home to diverse plant-based food commodities. The need to leverage the advantages of unprocessed/minimally processed foods (PFs) over ultra-processed foods (UPFs) is a system that requires exploitation. Most of the crops produced in the continent are either classified as traditionally or moderately PFs. However, the rise in industrialization and formalization of markets is impacting and marginalizing traditional food processing (FP). Current FP classification frameworks are briefly discussed. The level of processing of cereals, grains, fruits, vegetables, roots, and tuber crops in the continent requires intervention from nutritionists, food scientists, and scientific and governmental bodies to gain a holistic view and tackle the issue of food insecurity in Africa. This study reviews the levels of processing of African foods, challenges, and future directions.

## Introduction

Human foods can be classified based on the food type (i.e., plant- or animal-based), food groups (e.g., cereals, fruits, vegetables, roots and tubers, and bakery products), and level of processing. Food processing (FP) encompasses the sequence of unit operations any raw food material is subjected to, such as cleaning, cutting, crushing, milling, freezing, heating, and packaging, thereby leading to the physical and chemical transformation of the food from its natural state (1, 2). Foods are processed for preservation, shelf-life extension, safety, quality improvement, and sensory attributes (2, 3). FP is important in dietary needs as it provides consumers with safe foods with no harmful pathogenic microorganisms, reduced antinutritional compounds, and high functional, nutritional, and sensory properties. Processed foods (PFs) also offer convenience, a diversified diet, reduced preparation time, and constant supply to the consumer market during the off-season and adverse climate conditions, thereby guaranteeing regular supply to remote regions (4).

These methods and processes are designed to preserve natural foods, to make them suitable for more extended storage, and still fit for human consumption. Some minimally PFs are prepared and cooked as dishes or meals in kitchens at home or in restaurants or canteens combined with some PFs (5, 6). They vary in energy density and their content and balance of fats, carbohydrates, proteins and their fractions, vitamins, minerals, and other bioactive compounds (4). PFs are mainly processed and consumed as part of meals or dishes or may be used together with ultra-processed products to replace food-based freshly prepared dishes and meals (7, 8). Types of foods that are included in this group are canned or bottled vegetables and legumes (pulses) preserved in brine; peeled or sliced fruits preserved in syrup; tinned whole or pieces of fish kept in oil; salted nuts; reconstituted processed meats such as ham, ham bacon, and smoked fish; and cheese (9). Ultra-processed foods (UPFs) are commonly known as “highly processed foods,” which are produced and are added to some food items such as salt, sweeteners, or fat to include artificial colors and flavors, and preservatives that promote shelf-life, preserve texture, and increase palatability (10).

The continent of Africa is endowed with lots of plant-based foods that have been transformed into PFs over the years. Some of these foods are present in other continents, while others are specific, only to the African continent (11). Transformation of these plant products into shelf-stable food products is achieved through FP, leading to sustainable food systems for the continent (12). This study reviews the current FP classification systems, which category some African foods fall into, challenges, and future directions.

## Existing Food Classifications Based on Their Level of Processing

Eight FP classification frameworks identified are (i) Food Standards Australian New Zealand (FSANZ), (ii) International Food Policy and Research Institute (IFPRI), (iii) International Agency for Research on Cancer and European Prospective Investigation into Cancer and Nutrition (IARC-EPIC), (iv) National Institute of Public Health (NIPH), (v) NOVA, (vi) International Food and Information Council (IFIC), (vii) Poti, and (viii) Siga (13). As reviewed by Reardon et al. the FP evolution over the past five decades has seen a shifting trend from processing foods at home (traditional) to buying PFs and then preparing them at home (early to mid-transitional) to now eating out frequently (modern) (7). The intention of these classification frameworks was all epidemiological, except for Siga, whose intent was based on the development of food products and portfolios and to provide proper guidance to consumers on the overview of the food to help them make better choices (13, 14). The lowest level for all frameworks was unprocessed, and the highest level was UPF or highly PFs. Despite the different approaches of classification used by the frameworks, they all ultimately grouped foods as processed or unprocessed in a similar yet distinct manner. Several studies have explained and reviewed the various food classification frameworks (13–16). For up-to-date, detailed information on the conceptualization and challenges with the existing classification frameworks, refer to the study by Sadler et al. (13).

### Description of the Existing Food Classification Frameworks

The FSANZ method only classified foods as processed and unprocessed (Table 1), thus making the framework open to several interpretations. This was quite ambiguous (15, 17). The unprocessed class was not defined, while the processing was defined as treatments that caused significant changes to the food from its original state (15, 18). The IFPRI framework classified foods based on the degree of processing and was not elaborated. There were no definitions for the categories of unprocessed (e.g., fruits, nuts, fresh, and dried milk) and partially processed (e.g., lard, butter, and evaporated milk) foods. The PF category was defined as “*foods that have undergone secondary processing into readily edible form, likely to contain high levels of added sugars, fats or salt*.” Examples include patisserie and confectionaries. More emphasis was placed on industrial processing, while home processing was left out (13, 19). The NIPH framework encompasses unprocessed, locally made, non-industrialized vs. industrialized, and traditional vs. industrialized foods based on processing and temporality (19). The NOVA classification is a system of food classification based on the extent and purpose of their processing while considering the physical and chemical methods used for processing and the use of additives Monteiro et al. (8, 20, 21). This system places foods into four groups (13, 20, 22). NOVA group 1 is classified as unprocessed foods obtained directly from the plant or animal and have not been altered, which include grains, fresh fruits, and milk. The minimal level of processing of NOVA aims to preserve and extend the shelf-life of the food through washing, grating, freezing, crushing, and packaging (20). The foods in NOVA group 2 are processed culinary ingredients and derived from food group 1 through extraction, pressing, centrifugation, and mining processes. Examples include oils/fats, salt, and sugar. NOVA group 3 foods are called PFs and are created by combining food from groups 1 and 2 through industrial manufacturing processes such as canning, fermentation, and baking. Examples include unpackaged bread, cheese, and canned goods (vegetables/fruits/legumes). Finally, NOVA group 4 is called UPF. These are foods formulated by combining products from the other three groups through advanced industrial techniques such as extrusion. Examples include infant formula, reconstituted meat products, candies, and carbonated drinks (21).

**TABLE 1:** Food processing classification frameworks from around the world.

Classification frameworks	Definition of categories
FSANZ (Food Standards Australian New Zealand) Location: New Zealand (15, 18)	1. Unprocessed and minimally processed (not defined) 2. Processed foods (substantial change to the original state of the food)
IFPRI (International Food Policy Research Institute) Location: Guatemala (19)	1. Unprocessed (not defined) 2. Primary or partially processed (not defined) 3. Highly processed (secondarily processed into edible forms containing added salts, sugars and fats)
IARC-EPIC (International Agency for Research on Cancer–European Prospective Investigation into Cancer and Nutrition) Location: Europe (13, 15, 19, 23, 35)	1. Foods with an unknown process 2. Non-processed foods (consumed raw with no further processing) 3. Moderately processed foods 3.1 Modest processing, no further cooking 3.2 Cooked foods from raw or moderately processed foods 4. Highly processed foods 4.1. Processed staple/basic foods 4.2 highly processed foods.
NIPH (National Institute of Public Health) Location: Mexico (19)	1. Non-industrialized 1.1 Unprocessed 1.2 Locally made traditional foods 1.3 Traditional ready-to-eat foods outside the home. 1.4 Modern preparations outside the home 2. Industrialized traditional 3. Modern industrialized 3.1. Modern industrialized (single or mixed commercial products) 3.2. Industrialized traditional (up-scaled traditional Mexican foods)
NOVA Location: Brazil (8, 13, 15, 20, 21, 36, 37)	1. Unprocessed and minimally processed foods 2. Processed culinary ingredients 3. Processed food products (addition of salts, fats, sugar to products to make them tastier) 4. Ultra-processed products (contains no whole foods. Usually prepared from ingredients derived from foods.
IFIC (International Food Information Council) Location: United States (19, 24)	1. Minimally processed (require minimal processing such as packaging, grinding) 2. Processed for preservation 3. Mixtures of combined ingredients 3.1 Packaged mixes, jarred sauce 3.2 Mixtures, home-prepared 4. “Ready-to-eat” processed foods 4.1 Packaged ready-to-eat foods 4.2 Mixtures, store prepared 5. Prepared foods/meals
Poti Location: United States (13, 25, 26)	1. Less processed (unprocessed/minimally processed) 2. Basic processed 2.1 Processed basic ingredients 2.2 Processed for basic preservation or precooking 3. Moderately processed 3.1 Moderately processed for flavor 3.2 Moderately processed grain products 4. Highly processed 4.1 Highly processed ingredients 4.2 Highly processed standalone
Siga Location: France (13, 14, 27)	A. Un-/minimally processed A0. intact raw initial matrix A1. degraded basic matrix A2. culinary ingredients B. Processed B1. added salt, sugars, fat below official recommendations B2. added salt, sugars, fat above official recommendations C. Ultra-processed—loss of matrix/contain purified and denatured ingredient (excludes vitamins, minerals, tolerance of preservatives) C01. balanced nutritional profile & one industrial ingredient/additive (acceptable) C02. high added fat/sugar/salt C1. unprocessed industrial ingredients and/or limited additives C2. processed industrial ingredients and/or high additives C3. ultra-processed industrial ingredients and/or very high additives

The primary basis of the IARC-EPIC was the degree of processing at major comparison points such as raw vs. cooked, industrial vs. artisanal, and minimal vs. high processing. Minimal processing was not defined, but examples suggest that modest processing is “*close to the natural process*” (13, 23). The IFIC classification system defined FP as any intentional change to food outside its original derivation, based on the intricacies of processing with its accompanying physicochemical and organoleptic changes (24). This classification includes homemade foods at level 1 (minimally processed, e.g., homemade soup) or level 3 (mixtures of combined ingredients). The IFIC framework focuses on preserving the intrinsic properties of foods by comparing minimal processing vs. complex preparation and the level of value-added convenience (13).

The Poti classification system was developed by researchers at the University of North Carolina. It defined FP as any alteration of food from its natural state by the industry (13). The Poti framework has four classification levels based on the degree of industrial processing vs. convenience, the caloric content of each category in 12 years, and a comparison of the additives (e.g., sugar, fat, and sodium content) (25). They hypothesized that the nutritional quality of foods purchased in the supermarkets by American households might be due to a high correlation between the degree of FP and convenience. The system was developed by categorizing all bar-coded foods sold in supermarkets in the United States using product-specific ingredients and nutrients as markers (25, 26). Also, this study reveals that the United States market is dominated by highly processed and RTE foods with high sugar, fat, and sodium content over the periods assessed.

The Siga FP classification was formed to improve the NOVA framework. The Siga index classifies foods using the cumulative effect of a few factors such as the quantity, nature, function and degree of processing, and risk assessment of additives (sugar, salt, and fat addition) based on the scientific opinions of health agencies such as the WHO AND EFSA and the effect of these additives on the nutrient thresholds of food (27, 28). The Siga framework considered the degree of transformation of the ingredients and the loss of the “matrix” effect to achieve an even more holistic and realistic classification. This framework adopts a holistic approach instead of other frameworks where FP classification is reduced to just a sum of certain nutrients. This is termed a “reductionist approach” (29). They further explained that a sum of all the nutrients in the food matrix might have a more synergistic effect than just capitalizing on a few essential nutrients. They defined the holistic paradigm as “*an approach in food processing would lead technologists and food scientists to consider foods as systems that are not only a sum of their nutrients but rather a package of bioactive compounds included in a complex food structure.*” The Siga system contains four holistic groups and four reductionist subgroups based on the impact of processing on the food on the food matrix (14).

### Is There a Consensus Amongst the Classification Frameworks?

Crino et al. compared six frameworks, namely, FSANZ, Poti, IFC, IFPRI, NOVA, and IARC-EPIC. The authors tested 135 food categories of Euromonitor by applying the frameworks to several food types including packaged foods, detailing industrial FP specifically, and categorizing foods based on the levels of processing. A fundamental dichotomy of processed vs. unprocessed foods was noted for all frameworks, with several layers/levels occurring in the PF section. Their findings showed some similarities. However, the frameworks did not precisely match the PF category. The NOVA framework had the highest agreement with the other five (15). Some authors have argued that the NOVA system is not holistic because the aspect of food safety and domestic processing was not taken into consideration (4). In measuring the strength of the UNC, NOVA, and IFIC FP classification systems, Bleiweiss-Sande et al. assessed 100 foods consumed by children by comparing the three frameworks based on nutrient quality, inter-reliability, and similarity of the systems. Although a significant relationship was observed between nutrient content and processing category, they alluded that current classification systems may not be able to distinguish common foods consumed by children in the United States satisfactorily. This is partly due to the small scope of the study in terms of the number of systems and food mass studied (26). Due to the ambiguity caused by the different purposes of the various FP classifications, Martinez-Perez et al. hypothesized that the NOVA, IFIC, IARC-EPIC, and Poti classification frameworks would result in varying degrees of association between UPFs and cardiometabolic biomarkers. Their study showed a need to standardize the FP classification due to distinct differences in cardiometabolic biomarkers. However, all the assessed frameworks showed that UPF consumption negatively impacted nutrient quality (16).

Although the application of the NOVA classification showed a direct correlation between consumption of UPFs and metabolic diseases (30), these foods have been labeled bad due to high fat, sugar, sodium, energy density, and low dietary fiber and essential nutrients (8). This may be entirely unacceptable to categorize all UPFs as bad foods (14). A case in point is a study where 50 foods classified as UPFs were analyzed using the NOVA framework and European Regulation (EC) No 1924/2006 Nutrition Claims. No statistically significant direct relationships were found between the number of ingredients and energy, saturated fat, total sugar, sodium, AOAC fiber, and protein. The majority of UPFs identified had 60–80% less sugar, salt, and saturated fat and had 60% fiber and 30% protein. The author concluded that not all foods classified as UPFs are unhealthy (17). In fact, some UPFs are not health-friendly. However, all UPFs should not be discarded and labeled as evil because some raw materials must undergo ultra-processing before becoming edible (14, 17). A point in case is that unprocessed raw cassava contains hydrogen cyanide—a toxic compound that minimal processing may not remove, rendering it inedible (31). Therefore, processes such as fermentation, starch extraction, and grinding are necessary to transform the tuber into value-added products without depletion of nutrients (32). Another example is fresh milk which is not shelf-stable for long without processing to extend the shelf life or into other dairy products such as cheese, butter, and yoghurt (12). Consumers may be at risk of food infection without necessary food safety checks at home while handling fresh milk. Therefore, FP is required to provide consumers with safe, shelf-stable, and nutritious food. It is paramount to include home processing as a factor in classifying FP frameworks. It has been noted that there is a higher risk of food contamination due to poor handling and processing practices in the home environment (33). For instance, FP at home may pose more health risks than industrial PFs because the consumers’ addition of sugar and salt to food is not regulated. There is also the risk of pathogenic cross-contamination between foods.

The above examples show that we are still far from a one-size-fits-all system that can classify PFs due to the discrepancies amongst the frameworks (34). This is due to socio-cultural differences, intent, and goal of classification. The purpose of current and new classification systems that may be proposed in the future should be clearly defined with explicit examples. In addition, a holistic approach should be applied while classifying. This encompasses the entire steps across the food chain, including the processing extent place of processing (home vs. industrial), while also considering food waste reduction, food storage, transportation, environmental and epidemiological impact, and nutrition (17).

### Ultra-Processed Foods

Due to increased urbanization, there has been a sharp rise in the purchase of PFs in Africa over the last five decades (7). This has led to an increase in PFs in the form of ready-to-eat, ready-to-heat, and quick-cook foods requiring less preparation due to the “fast life” in urban regions compared with rural areas home-cooked meals that are still cherished (3). UPFs undergo several changes from their natural state. Raw agricultural commodities are introduced to different technological processes such as washing, cleaning, milling, cutting, chopping, heating, pasteurizing, blanching, cooking, canning, freezing, drying, dehydrating, mixing, packaging, or other procedures that alter the food from its natural state (38). Ingredients are also added, such as preservatives, storing, filtering, fermenting, extracting, concentrating, microwaving, and packaging (8). These products are characteristically ready-to-eat industrial formulations of cheap homogenized ingredients obtained from high-yield crops, notably sugars and syrups, refined starches, oils and fats, protein isolates, and sometimes from remnants of intensively reared animals. UPFs are produced to attract customers because they have a good appearance, smell, and better taste. The manufacturer uses sophisticated formulations of different food substances such as flavors, colors, emulsifiers, preservatives, sweeteners, thickeners, and other cosmetic additives (39).

According to the Siga framework, UPFs are either balanced (C01), greedy (C02), or processed to limit (C1, C2, C3). It is advised that the latter be avoided or reduced to occasional indulgence due to at-risk additives, which could be harmful to human health (28). However, some preservatives play a significant role in the promotion of food safety of food by preventing the growth of molds and bacteria. The most common preservatives used in the production of foods are ascorbic acid, sodium benzoate, potassium sorbate, tocopherols, and emulsifiers that prevent the separation of liquids and solids, e.g., soy lecithin monoglycerides. Examples of thickeners to add texture to foods are hydrocolloids such as xanthan gum, pectin, carrageenan, and guar gum (40, 41). Furthermore, food fortification is performed to produce nutritionally balanced UPFs where they were otherwise lacking. Fortified foods contain vitamins and minerals that are added after processing due to loss during processing, or they were added because they are lacking in the average diet (42). Mainly used fortificants are vitamin B (e.g., riboflavin, niacin, niacinamide, folate, or folic acid), beta carotene, iron (ferrous sulfate), vitamin C (ascorbic acid), vitamin D (42, 43), or amino acids to boost protein content (10).

Ultra-processing is characterized by different methods and ingredients to produce highly profitable branded products (44). UPFs are also available at low cost with a long shelf-life which is liable to displace the production and consumption of unprocessed or minimally PFs, PFs, and freshly prepared dishes and meals, or simply “real food” for short. UPFs are primarily formulated to increase human demands and cravings so that customers may enjoy eating them and can purchase more of such foods (44). Examples are sugary drinks, cookies, crackers, chips, breakfast cereals, frozen dinners, and luncheon meats, which minimally replace PFs in some consumers’ diets (21).

There are several disadvantages of consuming some UPFs that are related to human health, such as obesity, hypertension, cardiovascular diseases, dyslipidaemia, metabolic syndrome, gastrointestinal disorders, breast cancer, depression, and all-causes high death rate (14, 21, 28).

A global migration from indigenous and traditional food crops and agricultural production has changed the food scene in the last 50 years, especially in developing countries such as sub-Saharan Africa (Table 2). Statistical analysis of the world market showed higher consumption of UPFs in high-income countries such as the United States, Canada, the United Kingdom, and Australia. It indicated a rapid growth of UPFs in middle-income countries. Between 1998 and 2012, the market sales of sugary and salty snacks and soft drinks increased by 50% in upper-middle-income countries and more than 100% in lower-middle-income countries (21). Euromonitor statistical data showed that the per capita retail sales of three UPFs, namely, frozen products, snacks, and soft drinks in some low-middle income African countries (Cameroon, Egypt, Morocco, and Nigeria) increased by 180, 115, and 273%, respectively. In contrast, an increase of 129, 46, and 48% was reported in upper-middle African countries such as Algeria and South Africa (36, 45). This study concludes that the rise in the consumption of UPFs we see now is primarily influenced by the presence of industrial food manufacturing of UPFs, its retailing, and fast-food corporations (36). Most African countries, especially South Africa, have reported an increase in obesity in adults aged 18 years and above due to the consumption of UPFs (46–48). This is a direct result of the easy accessibility of UPFs on the shelves Hunter-Adams et al. (49). Most South African citizens eat more junk foods than indigenous minimal PFs (25, 43).

**TABLE 2:** Overview of 11 critical shifts in the 50-year evolution of processed food consumption in sub-Saharan Africa: who, what, when, where, and how.

Food products	Tradition	Early transition	Mid- to late transitional	Late transitional to early modern
Cereals consumed (not reflecting the form)	Home-produced millet, sorghum, and maize	Buy millet, sorghum, and maize; start buying rice and wheat	Buy more rice and wheat and more minor millet, sorghum, and maize.	Continue shift to rice and wheat.
Acquire minimally processed cereals	Pound grain at home	Custom mill flour or buy by scoop or large bag	Buy packaged branded maize flour and polished rice	Purchase highly and ultra-processed rather than minimally processed
Acquire minimally processed roots and tubers	Pound roots and tubers at home	Buy cassava flour by scoop or bag	Buy packaged cassava and yam flours	Continue shift to packaged cassava and yam flours
Acquire snacks and drinks	Cook and eat traditionally snacks and treats at home	Buy traditional snacks and treats	Buy ultra-processed packaged snacks and beverages	Increase purchases of ultra-processed snacks and SSBs
When snacks are consumed	Traditional festivals	Diverse special occasions	Weekly or daily	Increase frequency
Meal preparation and acquisition	Cook and eat meals at home	Buy traditional meals at a local street Vendors	Buy non-traditional meals at restaurants and street vendors	Buy at fast-food chains
Who buys meals away from home	No purchased m A few traditional snacks (fritters, mandazi) meals	Bachelors and students	Women and men working outside the home	Whole family
Purchases of highly processed foods	A few types (bread, mandazi)	A few traditional snacks (fritters, mandazi)	Many types	Increase diversity
Sources of processed Foods	Home	Small local retailers and neighbors	SMEs, stalls, and retailers in towns	Small shops and supermarkets

*Adapted from Reardon et al. (7).*

## FP in Africa

Traditional or indigenous crops play an important role as a symbol of heritage, trademark, and culture, besides offering an essential opportunity to diversify the food base through different ethnic groups (11, 12). Therefore, it is necessary to preserve diverse food practices, especially food preparation and consumption elements, as this knowledge can easily be lost over a few generations (2, 50). There is a significant risk that the knowledge around indigenous foods and potentially crucial ways of living more sustainably has already vanished (51). The African communities lose their identity of preserving those products that are easy to prepare because they are usually minimally processed, leading to food insecurity and livelihood in sub-Saharan Africa. Researchers argue that indigenous food plants played an essential role in the diet of African communities, the industrialization of food, and formalization of markets in countries such as South Africa have resulted in a decrease in the utilization of established domesticated wild plants and foods that had been stable for decades (11). The African traditional foods have been marginalized due to a lack of information on their use and importance in rural economies/cultures and their economic value. Moreover, there are limited reliable methods for measuring their contribution to farm households and the rural economy; lack of world markets (except for a handful of products); irregularities in supply; quality standards; and storage and processing technology (52). Traditional foods that were consumed back then have more nutritional values because some of them were consumed without adding salt, sugar, oil, and other food additives, which are reported to cause some chronic diseases. Many modifications are done to the indigenous crops for converting them into value-added products that increase the availability of products in large quantities to reduce food security. The major problem with the value-added product is the increased harmful diseases caused by the substances added to the final product (11, 12, 52). This section reviewed some food groups (e.g., cereals and grains, fruits and vegetables, and roots and tuber crops) specific to the African continent and their reported level of processing.

### Cereals and Grains

**FIGURE 1:**
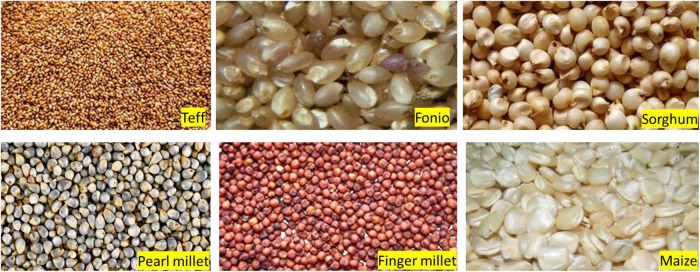
Cereals and grains of Africa. (https://www.google.com/search?q=cereal+grains&sxsrf=AOaemvL2cmQ98ztkUbsjOzKZQi6eK6n95A:1638149496818&source=lnms&tbm=isch&sa=X&ved=2ahUKEwiryeK2trz0AhUcQUEAHf4CDsYQ_AUoAXoECAEQAw&biw=1366&bih=657&dpr=1.

Cereals and grains form the more significant percentage of food consumed worldwide as they are excellent energy sources. The various cereals grown in Africa include wheat, millet, maise, fonio, teff, sorghum, and rice (Figure 1). Grains require various levels of processing to transform them into edible products. Since it is the source of carbohydrate foods consumed by most people, several bakery products have been formulated from cereals (Table 3). Several research studies have been conducted on cereal grain processing (42, 53, 54). According to the Siga framework (Table 1), the level of processing has been assigned to the foods in Table 3.

**TABLE 3:** Classification of African foods based on their level of processing based on the Siga food processing index.

Group	Area of distribution	Food products and Siga index assignment	References
Cereals and grains		Unprocessed grain^1^, broken grains^2^, flour^2^, breakfast cereal^7^, gruel^3^, pastry^7^, flatbread^3^, muffin^7^, smoothie powder^4^, infant food^3^.	(42, 53, 54)
Millet	WA, SA, CA		
Sorghum	WA, SA, EA		
Fonio	WA		
Teff	EA		
**Tubers**			
Yam	WA	Boiled yam^2^, yam flour^2^, noodles^7^, bread^7^, chips^6^, crisps^6^, *amala*^3^,	(55, 56)
Cassava	WA, CA	Dried cassava chips^2^, boiled^2^, starch, stiff porridge^3^, composite flour, biscuits^7^, muffin^7^, biscuits^7^, bread^7^, *fufu*^3^, *gari*	(31, 57)
Cocoyam/Taro	WA, SA	Dried or fried chips^7^, boiled taro^2^, starch^2^, flour^2^, cake^7^, bread^7^, cookies^7^, doughnuts^7^	(58–61)
African Yam bean (AYB) tuber		Yoghurt^4^, cookies^7^, *fufu*^3^, breakfast cereal^7^	(62, 63)
**Fruits and vegetables**			
African bush mango	CA, humid WA	Juice^2, 3^, jam^3^, wine, dried fruit^2,3^, raw fruit^1^, dried fruit powder, seed flour, extracts, ice cream	(64, 65)
African pear	CA, humid WA	Raw^1^, boiled^2^, roasted^2^, oil, essential oils^2^	(66, 67)
Baobab fruit	EA, WA, CA	Pulp powder^2^, juice^3^, flavouring^6^	(68–70)
Baobab leaves		Cooked^2^, dried leaves as a condiment and sauce garnishes^2^	(71)
AYB seeds		Cooked legume^2^, flour^2^, food fortificant^2^	(72)

*EA, WA, CA, and SA represent Eastern, Western, Central, and Southern Africa, 1—A0, 2—A1 and A2, 3—B1, 4—B2, 5—C01, 6—C02, and 7—C1, C2, and C3 of the Siga framework described in Table 1.*

### Roots and Tubers

Root and tuber crops supply energy and nutrition to over two billion people and are an essential income source for farmers in rural and marginalized communities. Root and tuber crops are economically versatile, providing cash, food security, and regular food crops. The waste products (peels) can be used as industrial raw materials and livestock feed (73). The tubers discussed in this section are grown and consumed on the African continent and their normal processing levels.

#### African Yam Bean Tuber

**FIGURE 2:**
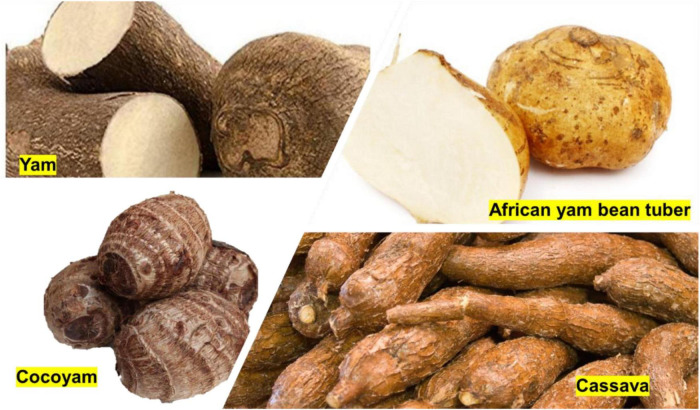
African tuber crops (https://www.google.com/search?q=tuber+crops+in+africa&sxsrf=AOaemvIjAfPFMI4V4BheAZXga3ORPC33wQ:1637923213131&source=lnms&tbm=isch&sa=X&ved=2ahUKEwjPlqa667~×~0AhWB87sIHcIoBp8Q_AUoAXoECAEQAw&biw=1366&bih=657&dpr=1).

African yam bean (AYB) (*Sphenostylis stenocarpa*) plant is mainly grown and consumed in West, Central, and East Africa (Figure 2). It is a potential food security crop that is versatile due to its edible seed and roots George, Ajibola (48, 50, 72, 74). Although regarded as an orphan crop, this plant is suitable for potential food security in Africa for the following reasons: it survives in broad climatic conditions, contains essential nutrients, has a cultural link to Africa, and provides the potential for food-to-food fortification of staple foods Ojuederie, Teye (49, 51, 73, 75). AYB tuber has superior protein content (15%) compared with cassava and yam at < 2%, thus making it a valuable tuber for the development of protein-rich food products Ojuederie (49, 73). The starch of AYB tuber and seed showed superior thermal properties and were resistant to amylolytic enzymes indicating its potential use for the development of low Glycemic index (GI) foods Malumba (52, 76).

The AYB seed is helpful for food fortification to compensate for nutrient loss in foods due to processing. Its seeds are primarily consumed in Nigeria, West Africa. This is because the proteins (20–30%), carbohydrates (50–64%), dietary fibers (3–10%), total minerals (2–5%), and amino acid contents compare favorably with other legumes such as soybean. AYB seed starch has been extracted and shown to possess superior functional properties (76). The seeds are usually boiled with salt, pepper, and palm oil and consumed as a bean soup. Recently, other products developed from AYB seed include a yoghurt-like product (77), biscuits (78), breakfast cereal (76, 79), noodles (80), and cassava enriched *fufu* (81).

#### Cassava (*Manihot esculenta*)

The highest production of cassava (*Manihot esculenta*) in Africa is in Nigeria—accounting for 19% of the total world’s population, followed by other non-African countries such as Thailand (11%), Indonesia (9%), and Brazil (8%). This tuber crop offers a potential income stream for exportation. Still, due to problems such as weak trade, transport routes, market access, and quick deterioration of the tuber, most of the cassava produced on the continent is consumed in Africa (82). Cassava-based products are primarily consumed in traditional forms, such as fermented cassava granules (*garri*) and stiff porridge (*fufu, eba*), retaining their essential nutrients. More recently, cassava flour has been used alone and composited with other flours to produce biscuits (82), bread (83, 84), muffins (85), Dewi et al. (86), Ramírez et al. (87), and pasta (88). However, these ultra-processed products are not yet widespread or available on the shelves; hence, there is a need for more commercialisation of cassava flour on the continent and the exportation of these products within and outside of the African continent.

#### Taro Root

Cocoyam (*Colocasia esculenta* [L.] Schott) is a tropical root crop that originated in Asia and spread to the rest of the world. The global production statistics show that 69.42% is cultivated in Africa, with Nigeria ranking the highest at 27.14%. Surprisingly, none of the African countries cultivating taro make it to the top ten exporters (84). This is a saddening gap in economic turnover for cocoyam farmers in Africa. The two species extensively grown in Africa belong to the Araceae family, namely, *Xanthosoma sagittifolium* and *C. esculenta*. Despite its long-standing existence, it is marginalized in food production, export, and industrial usage. However, that narrative is changing as scientific investigations into its utilisation as a food fortificant (59, 60, 89, 90) and food ingredient (91–94) are currently underway. Nutritionally, taro is superior to cassava and potatoes in protein (11% dry weight) and carbohydrate (87% dry weight) contents. The corm of taro root has been consumed over the years because the starch granule of taro is small and easily digestible compared with other tubers. This makes it an ideal source of carbohydrates for people with digestive problems, especially the elderly (90, 95, 96).

### Fruits and Vegetables

**FIGURE 3:**
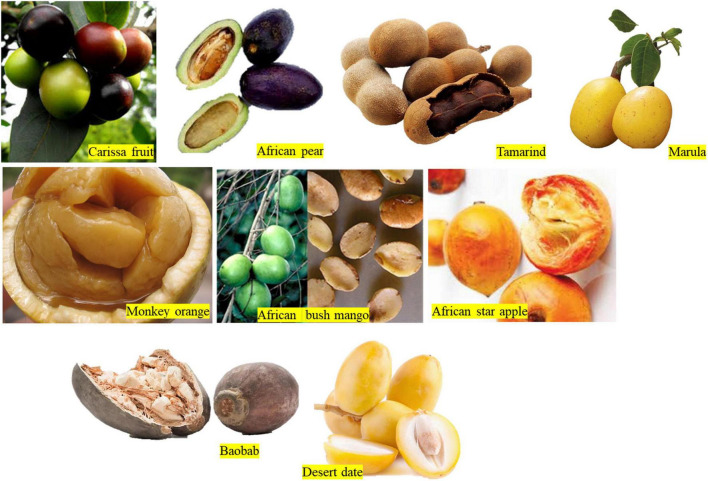
African native fruits (https://www.google.com/search?q=fruits+in+africa&sxsrf=AOaemvID5L7IffILh1Ps_tXHgaANzaTqpA:1638116809389&source=lnms&tbm=isch&sa=X&ved=2ahUKEwjcipjUvLv0AhUMKewKHQs_CnYQ_AUoAXoECAEQAw).

Apart from the known commercial fruits in the market such as orange, mango, apple, pear, and grapes, numerous indigenous fruits have not been exploited for food security in Africa. Some of these fruits were adequately reviewed by Stadlmayr et al. (68). Examples of the indigenous fruits of Africa (Figure 3) are *Adansonia digitata* L., *Balanites aegyptiaca* (L.) Delile, *Dacryodes edulis* (G. Don) H. J. Lam, *Irvingia gabonensis*, *Sclerocarya birrea, Syzygium guineense* (Willd.) DC., *Tamarindus indica* L., *Uapaca kirkiana* Müll. Arg., *Vitex doniana* Sweet, *Ziziphus mauritiana* Lam., and *Chrysophyllum albidum* (68). The focus of this section is to bring to the limelight the underutilized African fruits and vegetables and their levels of processing.

#### African Bush Mango (*Irvingia gabonensis*)

*Irvingia gabonensis* has a morphology like that of mango, hence the name “African bush mango.” Other names include *bush mango, dika nut, odika, ogbono, manguier sauvage*, or *chocolatier* (65). It is indigenous to the humid forest zone of some African countries such as Congo, Uganda, Nigeria, Angola, and the Ivory Coast. It has an edible fruit pulp and an oil-rich kernel enclosed in a hard stony nut. It is widely used as food and medicine. The fruit pulp and seed (Figure 3) have gained prominence in pharmaceutical weight loss supplements in the United States (97). There are two species common in West Africa. The sweet, yellow-fleshed edible fruit (*Irvingia gabonensis*) and the one that is processed for cooking are characterized by a bitter and non-edible mesocarp (*Irvingia wobolu*). The vitamin C (51–76 mg/100 g) content of *I. gabonensis* fruit is higher than the common mango (40 mg/100 g). The ripe and unripe bush mango fruit has a shelf-life of 2 and 10 days, respectively. Therefore, processing is highly needed to prevent postharvest losses. The level of processing of the fruit is minimal to average ranging from drying, grinding, pressing, and fermentation. These have yielded processed products such as juice, jam, wine, dried fruits, and dried fruit powder (64, 65, 98).

#### African Pear (*D. edulis*)

**FIGURE 4:**
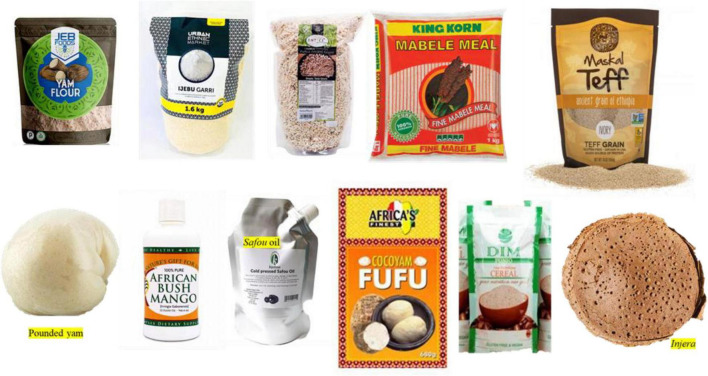
Some African processed foods (https://www.google.com/search?q=african+processed+foods&rlz=1C1CHBF_enZA990ZA990&sxsrf=APq-WBtQl6wnr_h-2NX_pG9IAOcEX6L9_g:1647524662762&source=lnms&tbm=isch&sa=X&ved=2ahUKEwj09qDSo832AhUBgFwKHeIeCDEQ_AUoAXoECAEQAw&biw=1366&bih=657&dpr=1).

*Dacryodes edulis*, also known as butter fruit, bush pear, and African plum, is a fruit tree native to West African countries. Its local name differs in different countries. It matures into a pink olive-like fruit and turns dark purple upon ripening. It is called *ube* in Nigeria, *atanga* in Gabon, and *safou* in Cameroon (67). *D. edulis* is a highly underutilized tropical crop. The fruit is usually eaten raw as a snack, and it can also be roasted or boiled as side dishes. The fruit pulp has a rich lipid content accounting for 72.6% of the whole fruit, 44% of the pulp, and 27.3% of the seed and fatty acids up to 60% oleic and palmitic acid (66). The protein content (34% in the seed and 26% in pulp) of *D. edulis* is superior to soybean (14%). Essential oils such as α-pinene α-phellandrene, limonene, and β-pinene have been extracted from the resin, fruit, and seed of *D. edulis* for medicinal use (99). The fruit is high in calcium, potassium, and ascorbic acid. The array of nutrients in *D. edulis* makes it a “superfood” and a potential food security crop to combat malnutrition in the malnourished population of Africa. Cold-pressed *D. edulis* oil made in Africa is available on market shelves (Figure 4).

#### Baobab Fruit and Leaves (*A. digitata* L.)

*Adansonia digitata* L., known as baobab in English, is naturally distributed in Eastern, Western, and Southern Africa (68). Baobab is used as food, medicine, and animal feed. Every part of the baobab plant, i.e., bark, leaves, flower, root, fruit pulp, and seeds, is edible (100). African names for baobab are *isimuhu* (South Africa), *kouka* (Nigeria), *sira* (Mali), *mwambo* (Kenya), and *mlonje* (Malawi) (101). The fruit pulp is said to have antioxidant, anti-inflammatory, anti-microbial, and analgesic properties. The powder is derived from the fruit pulp and used in various ways such as adding to maize gruel, mixing with water as juice, and using in fermentation (69, 70). Due to high levels of anti-nutrient compounds such as tannins and phytic acid, the processing of baobab fruit into a value-added product is necessary. Even though the baobab is classified as the fruit of Africa, the leaves are often overlooked. Baobab leaves can be cooked just like any green vegetable in season and dried, ground into powder, and used as food condiments during off-seasons. The leaves are rich in iron, vitamin A, and protein (71).

## Future Directions

There are increasing global efforts, substantial interest, and scientific research on plant foods from sub-Saharan Africa. A lot of research still needs to be performed on the entire value chain of farms to consumers. Further research is required for all aspects of the plant crop to optimize its benefits and valorization. Most plant-based foods on the African continent are still minimally prepared in their natural state, therefore, offering the benefits of whole foods—supporting the holism paradigm. However, the challenges that plague the low-income countries of Africa, such as unreliable road networks, poor storage infrastructures, and electricity issues, most foods are prone to spoilage. Therefore, the level of processing that ensures nutritional balance and fewer additives and promotes less wastage with a minimal or no negative environmental impact is encouraged. Future directions range from the engagement of policymakers to advancing scientific understanding using the various technologies of the fourth industrial revolution, including green technologies enabling maximum utilization of the different underutilized crops in addressing global food security and nutrition. Intensifying research on plant foods of Africa brings these crops at par with socioeconomic and scientific knowledge as crops from other parts of the world. The following areas, among others, are recommended for a balanced FP in Africa: (a) scoring foods in a hierarchy where a holistic index is first applied, followed by a compositional index, avoiding excessive valorization of UPFs, (b) understanding plant starch chemistry involving composition, isolation, physicochemical properties, and starch modification methods in search of novel properties and the application of modified starch in food systems, (c) modification of plant proteins for improved functionality highlights that although, through minimal physical, mechanical, and biological techniques are widely being adapted to produce a functional ingredient such as texturized vegetable proteins, hydrolyzed vegetable protein, clean label protein concentrates, de-flavored protein isolates, protein flour, and grits, and (d) promotion of holistic approach in line with the Siga framework.

## Author Contributions

AIOJ conceptualized the manuscript. OOO crafted the outline and led the manuscript writing. SER, OOO, and AIOJ wrote different portions of the manuscript. OOO formatted the final version of the manuscript. AIOJ reviewed the manuscript. All authors approved the submitted version of the manuscript.

## Conflict of Interest

AIOJ is currently engaged with VicFame Pty Ltd. The remaining authors declare that the research was conducted in the absence of any commercial or financial relationships that could be construed as a potential conflict of interest.

## Publisher’s Note

All claims expressed in this article are solely those of the authors and do not necessarily represent those of their affiliated organizations, or those of the publisher, the editors and the reviewers. Any product that may be evaluated in this article, or claim that may be made by its manufacturer, is not guaranteed or endorsed by the publisher.
